# A Review of Robot-Assisted Lower-Limb Stroke Therapy: Unexplored Paths and Future Directions in Gait Rehabilitation

**DOI:** 10.3389/fnbot.2020.00019

**Published:** 2020-04-15

**Authors:** Bradley Hobbs, Panagiotis Artemiadis

**Affiliations:** Human-Oriented Robotics and Control Laboratory, Department of Mechanical Engineering, University of Delaware, Newark, DE, United States

**Keywords:** gait rehabilitation, rehabilitation robotics, review, stroke therapy, therapeutic devices

## Abstract

Stroke affects one out of every six people on Earth. Approximately 90% of stroke survivors have some functional disability with mobility being a major impairment, which not only affects important daily activities but also increases the likelihood of falling. Originally intended to supplement traditional post-stroke gait rehabilitation, robotic systems have gained remarkable attention in recent years as a tool to decrease the strain on physical therapists while increasing the precision and repeatability of the therapy. While some of the current methods for robot-assisted rehabilitation have had many positive and promising outcomes, there is moderate evidence of improvement in walking and motor recovery using robotic devices compared to traditional practice. In order to better understand how and where robot-assisted rehabilitation has been effective, it is imperative to identify the main *schools of thought* that have prevailed. This review intends to observe those perspectives through three different lenses: the goal and type of interaction, the physical implementation, and the sensorimotor pathways targeted by robotic devices. The ways that researchers approach the problem of restoring gait function are grouped together in an intuitive way. Seeing robot-assisted rehabilitation in this unique light can naturally provoke the development of new directions to potentially fill the current research gaps and eventually discover more effective ways to provide therapy. In particular, the idea of utilizing the human inter-limb coordination mechanisms is brought up as an especially promising area for rehabilitation and is extensively discussed.

## 1. Introduction

Stroke is typically caused by a long-term lack of oxygen to the brain through a blood vessel bursting or clotting. Since this event usually occurs on one side of the brain, the effects are generally seen on the contralateral half of the body in the form of hemiparesis. This partial paralysis is common after stroke and makes a significant impact on daily life. After initial onset, recovery in the early stages is crucial to mitigate the long-term effects of stroke. More people are in need of stroke rehabilitation every year, and the cost for post-stroke patients with a need for continuous care is still high and projected to substantially increase in the next decade (Benjamin et al., 2019). In order to reduce the cost and increase the efficacy of post-stroke rehabilitation, it is crucial to determine and use the methods that prove to provide the best outcomes.

In recent years, robotic and electromechanical systems have gained increased interest in the rehabilitation community for their ability to automate the tedious and time intensive therapy needed for beneficial patient outcomes (Sale et al., 2012; Calabrò et al., 2016). Because locomotion is the result of complex dynamic interactions between feedback mechanisms and a central controller in the brain, the rehabilitation methods that work the best use a fundamental understanding of this coordination of human gait (Gassert and Dietz, 2018). It is well-known that in order to be effective, therapy should begin as soon as possible and provide an intensive training that incorporates multiple sensory mechanisms in a structured way (Poli et al., 2013). Robotic and electromechanical systems for rehabilitation purposes are designed with the intent of evoking the muscle activation synergies and neural plasticity through specific repetitive motor coordination exercises. Because brain tissue cannot simply be repaired in the exact way as before the damage, in order to regain a physical ability such as walking, the brain must be *rewired* along intact, active neural pathways. This influences therapies that incorporate various sensory inputs, experiences, learning, and especially motor training (Poli et al., 2013), showing there is a link between vigorous multisensory rehabilitation and recovery in stroke patients. Therefore, neural pathways that are not normally in use might be triggered to make up for the lost pathways. The intensity of stimulating those pathways can be drastically increased by introducing robotic devices to aid the physical therapists.

Because of the fast pace in which rehabilitation robotics has grown, robots and autonomous systems are longing to be the standard in rehabilitation. Due to both a rapid increase in technological improvements (Reinkensmeyer et al., 2004; Schmidt et al., 2005b; Hogan et al., 2006; Johnson, 2006; Patton et al., 2006) and a rapid increase in neurological understanding of rehabilitation (Kwakkel et al., 2008; Carter et al., 2010; Albert and Kesselring, 2012), there is a need to summarize where we are currently at with popular and emerging methods. This paper is an attempt to organize and categorize the ways in which we think about stroke rehabilitation, in order to produce more effective approaches to be developed in the future, while making sure to learn from past mistakes.

**Figure 1:**
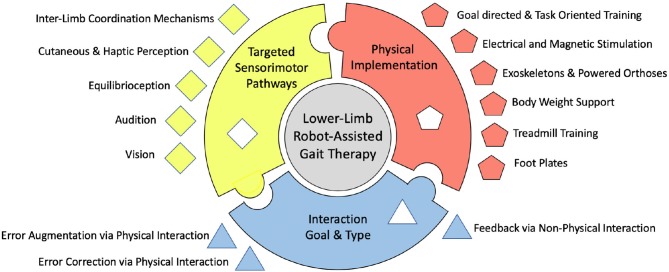
Depiction of the proposed organizational chart of existing robot-assisted stroke rehabilitation methods.

Moreover, there is a significant disparity between engineers that create devices for rehabilitation, and the underlying neuroscience related to motor deficits and rehabilitation after stroke. While this gap is certainly closing, it can be further bridged by understanding the underlying mechanisms for gait, gait adaptation, and gait therapy and by connecting promising technological advances in robotics with promising, related underlying neural pathways. Many of the studies and methods shown in this paper have produced promising results, but the proof of long-term benefits is required for the proper use of the word rehabilitation. The critical difference of this paper compared to previous reviews (Dickstein, 2008; Vallery et al., 2008; Marchal-Crespo and Reinkensmeyer, 2009; Schwartz et al., 2009; Díaz et al., 2011; Horno et al., 2011; Morone et al., 2011; Conesa et al., 2012; Mehrholz and Pohl, 2012; Pennycott et al., 2012; Chang and Kim, 2013; Kelley et al., 2013; Viteckova et al., 2013; Waldner et al., 2013; Zhang et al., 2013; Swinnen et al., 2014; Venkatakrishnan et al., 2014; Mehrholz et al., 2017; Agostini et al., 2018; Bruni et al., 2018), is that each individual point of view behind the creation of these methods is grouped into schools of thought, or approaches, based on a fundamental understanding of rehabilitation. This paper systematically reviews the different methods used by scientists to study and rehabilitate gait in humans and discusses the gaps in research that have yet to be filled, prompting potential new directions in the field. These schools of thought are the desired goal and type of interaction, the physical implementation of the method, and the neural mechanisms that are intended to be targeted or evoked, as depicted in Figure 1. There are many different tools and ways of thinking about gait rehabilitation, so within each school of thought, some of the gaps left behind are put forth.

## 2. Interaction Goal and Type

Each rehabilitation technique and system known today can fall under many different categories depending on differing points of view. One distinction can be made in the area of how the method will interact with the subject based on the goal of the therapy. For example, the most popular developments use some sort of physical connection between the subject and the machine. Furthermore, there is another distinction made in the objective of this interaction. In all cases, this physical interaction can be grouped according to if the interaction is intended to correct the subject's pathological gait, or if it is intended to perturb, or induce error into the gait. There has also been some research into methods that do not directly interact with the patient's gait in a physical way, but offer a form of rehabilitation in a strictly informational or communicative way. The majority of the current methods should fit into these three ways of thinking sub-categories, as it is further discussed below.

### 2.1. Error Correction via Physical Interaction

The human gait is often thought to be the result of complex sensori-motor neuro-mechanical systems that use real-time feedback to control the different muscle groups. The main objective of this controller is to produce a steady state walking pattern, considering any errors produced by the internal sources such as muscle spasm or overshoot, and external sources such as ground stiffness changes or interaction forces. In patients with post-stroke hemiplegia, there is a loss of coordination in muscle activations in certain muscle groups, or synergies on one side of the body. This causes the gait patterns in those individuals to exhibit common undesired traits such as drop-foot (Krebs et al., 2008). An idea that has been proposed in the past is to try to minimize those undesired activation patterns through robot-assisted rehabilitation. The long-term objective of the robotic intervention in that framework is to minimize the difference (error) between the normal and the paretic movement of the limb, while increasing repeatability and intensity of training. In general, this is through augmenting and precisely automating movements that would normally be followed through manually by a physical therapist. Thus, use of a robotic system minimizes therapist fatigue and increases repeatability (Freivogel et al., 2009; Peurala et al., 2009).

Early methods developed were in the form of systems intended to allow for prolonged training sessions and reduced therapist workload by automating the process of facilitating gait patterns (Colombo et al., 2000, 2001; Belforte et al., 2001). Many researchers use a trajectory tracking based approach to gait training (Beyl et al., 2008). A robot with upper and lower limb connections that allowed for walking velocity updates through generated spatial motions on the sagittal plane for each foot was developed by authors in Emken et al. (2005). For ankle assistive devices, authors in Agrawal et al. (2005) developed an ankle-foot orthosis to assist the tibialis anterior muscle in maintaining proper foot position for subjects with ankle flexion/extension control and inversion/eversion control. To avoid imposing constraints on naturalistic walking due to a robot's kinematic structure, the work in Aoyagi et al. (2007) suggests assisting the pelvic motion during stepping, and providing a type of compliant assistance to avoid perturbed rather than assisted stepping. This was intended to be a compliant robot that could act either in aid of the trainers, or in place of them if desired, and tailored the desired trajectory for each subject. Authors in Bharadwaj et al. (2005) and Bharadwaj and Sugar (2006) also emphasized repetitive task training as an effective form of rehabilitation for people suffering from stroke and presented an ankle rehabilitation method based on a tripod mechanism which moves the ankle in dorsiflexion/plantarflexion and inversion/eversion.

Some implementations for the seated position were presented in Bouri et al. (2009) and Chisholm et al. (2014) and then for the standing position through a deambulator mechanically interfaced with the verticalized orthoses (Bouri et al., 2006). Many methods use velocity or moment control (Chen et al., 2009) or an idea of feedback control of joint trajectories through modulated friction brakes (Farris et al., 2009a), which are used in conjunction with electrical stimulation. This unidirectionally couples hip to knee flexion and aids hip and knee flexion with a spring assist (Farris et al., 2009b). Specifically used for stair ascent and descent, powered assistance in the sagittal plane at both hip and knee joints and can be used in conjunction with an ankle foot orthosis (Farris et al., 2012) or functional electrical stimulation (FES) (Ha et al., 2012, 2016). Studies suggest walking with error correcting devices such as an exoskeleton provides increase in walking speed and a concomitant decrease in required exertion relative to walking with other knee-ankle-foot-orthoses (Farris et al., 2014).

Another ankle robot (Forrester et al., 2013) uses an internal model-based adaptive controller that both accommodates individual deficit severities and adapts to changes in patient performance. In general, the main purpose of an ankle-based system is to prevent slapping the foot after heel strike, and to control the ankle joint to actively minimize the fore foot collision with the ground (Hwang et al., 2006). This can be achieved by lifting the foot during swing but supporting further gait movements by controlling of the center of mass (Hesse et al., 2000). This system was later adapted to simulate level floor walking as well as climbing up and down stairs (Hesse et al., 2010). These systems intend to simulate gait-like movement through simulating stance and swing phases.

### 2.2. Error Augmentation via Physical Interaction

Many researchers have approached rehabilitation with the notion of a subject learning his or her own walking pattern through unexpected physical contact made to him or her, which elicits a reaction response in order to correct for the disturbance. It has been shown that individuals with cerebral damage from stroke have a normal capacity to make both reactive and predictive locomotor adaptations during walking (Choi and Bastian, 2007). The idea here is that neural plasticity is evoked through the brain attempting the gait correction in response to the disturbance. The brain perceives the error through various senses and neural pathways in the body and corrects based on this feedback. Typically, this still involves repetitive and frequent trials in order for a long-term effect to be realized in many cases.

An early example of this interaction method is the work presented in Girone et al. (2001), where a Stewart platform supplies resistive forces in response to virtual reality-based exercises. Error inducing methods are meant to manipulate human stepping, but can be used to study the mechanical properties of different joints as well (Roy et al., 2007). Another early implementation that emphasizes back-driveability and force generation capability shows that this method can induce motor adaptation and long-term after-effects (Reinkensmeyer et al., 2003). Many robotic tools provide different assistance levels, but also may have modes that challenge the subject's posture (Peshkin et al., 2005), elicit a stumbling like response (Schmidt et al., 2005a), regulate force feedback (Barkan et al., 2014), or induce perturbations (Schmidt and Werner, 2007) and resistance (Saglia et al., 2009; Klarner, 2010). In some cases, force-field-based perturbations can cause a subject to adapt to the applied field and follow normal gait pattern until it is turned off (Koopman et al., 2013). In other cases, these perturbation-based methods attempt to induce error by unexpectedly removing the perturbations and observing the after-effects (Reinkensmeyer et al., 2014), during treadmill training (Skidmore et al., 2015) or over-ground walking (Martelli et al., 2019). The idea of augmenting the error feedback is also shown to reduce some asymmetries in gait (Bishop et al., 2017). Even after therapeutic intervention, counteracting force perturbations can lead to improved responses for real-world loss of balance in regular life (Matjačić et al., 2018) by applying these force perturbations in a controlled setting (Olenšek et al., 2018).

### 2.3. Feedback via Non-physical Interaction

The third distinction is made for methods of rehabilitation that do not directly interact with the subject in a physical manner. This means that there are no corrections or perturbations evoked directly or indirectly through the senses. This is much less common but is emerging and can have the benefit of a greater patient independence. One of the ways to do this is by having a socially assistive robot that will give some sort of informational feedback to the subject through audio or visual means (Matarić et al., 2007). While this informational feedback is often coupled with physical contact in some way, it is worth mentioning briefly on the aspects of the non-physical method. Self-training is a relatively new method for rehabilitation that is augmented with robotic assistants that guide and observe patients during tasks (Gross et al., 2014). This comes with the challenge of the navigation and perception of humans and human behavior (Losey and O'Malley, 2019). Described as socially assistive, these robotic platforms describe a modern thought process for the role of robots in stroke therapy for survivors that have standing and walking mobility (Feil-Seifer and Matarić, 2005).

Another non-physical method for rehabilitation could be communicating information to the subject about how the subject is progressing in terms of his or her gait by making gait data accessible for self-correction. This informational feedback can be data provided to a subject to influence the rehabilitation process in some way. The distinction is made when the information is in the form of meaningful data that the subject can use in independent, self-driven rehabilitation, potentially post-therapy. This can include displaying movement patterns throughout the day and comparing with daily goals or informing the overall physical progress statistics of the subject. To achieve improvements in all phases of therapy, supplementing the patient with his or her own data is a potential addition in stroke therapy.

## 3. Physical Implementation

The second of the three categories that this paper uses for grouping the robot-assisted gait rehabilitation approaches, is the physical way in which each method is implemented. Even if the type of interaction is non-physical, the implementation of this method still has a physical attribute. Any robot-assisted rehabilitation method should fit into one of the following physical implementations. Many of these implementations assist physical therapists either in determining progress or in alleviating the strain of high intensity and repetitions by automating the therapy process. It should be noted that one physical implementation can use more than one idea from the Type and Goal of Interaction subcategory; consequently, this is where many gaps can be found.

### 3.1. Body Weight Support

Body weight supported training has been used in many studies in the past. Typically coupled with a treadmill, a body weight support allows the therapist to take a varying degree of weight off of the subject (Bouri et al., 2006; Seo and Lee, 2009). This is often used to minimize the effects of balance impairments or when the patient is unable to independently support his or her own weight (Stauffer, 2009). Perhaps the earliest form of mechanically augmented rehabilitation is the use of an over-ground body weight support (Hesse et al., 1999) as opposed to other techniques that may use body weight support in treadmill training. This includes a body weight support system that is connected to a base with wheels for mobility when a treadmill may not be desired (Peshkin et al., 2005). In general, proper body weight support is provided to reduce the balance and postural control mechanisms for both treadmill (Skidmore et al., 2014) and over ground studies (Peshkin et al., 2005).

### 3.2. Foot Plates

Many early physical approaches use only a foot attachment for rehabilitation and for exercise studies (Homma and Usuba, 2007). This has the benefit of being applied while a patient is in the early stages of stroke who may be bedridden (Monaco et al., 2009) or wheelchair bound (Hesse and Werner, 2009). Early systems may call these “haptic interfaces” (Girone et al., 2001), however this term is too broad for modern robotic classifications. With physical therapist assistance, high intensity training with these can lead to better gait ability (Hoölig et al., 2007). These can also apply assistive and resistive training (Saglia et al., 2009). The foot plate approach differs from other robotic methods in that only the foot is attached to the external device (Schmidt et al., 2007). This can have the impression of acting as a robot that actuates the foot in a Cartesian space, rather than in a joint space (Tsoi and Xie, 2008). These are typically actuated to move the foot into a trajectory (Freivogel et al., 2008) but can also perturb the normal walking pattern by using error inducing ideas. Since only the interaction with the foot is controlled, the foot plates have the advantage of being able to simulate different walking surfaces such as stairs (Hesse et al., 2000, 2010; Yano et al., 2010), inclines and uneven surfaces (Iwata et al., 2002). Some foot plates are also in the form of stationary platforms that allow the patient to sit (Bouri et al., 2009) or stand (Boian et al., 2005) in place and can aid in balance (Ding et al., 2010). Individuals who are not able to support their full body weight onto the legs benefit from the foot plate approach.

### 3.3. Exoskeletons and Powered Orthoses

The largest subcategory and the one that has received the most attention in the area of rehabilitation for many neurological disabilities including stroke is the exoskeletons and powered orthoses category. Many of the ways rehabilitation attempts are made is through an exoskeleton or a powered orthosis. These attach to the leg of the subject and induce an actuated torque directly on one or more of the joints (Veneman et al., 2007). This is most often used in conjunction with motors and some form of a controller to actively provide precise joint torques. Depending on the desired interaction goal, these can be used over ground (Murray et al., 2014) or in conjunction with a treadmill (Jezernik et al., 2003), and can have improved functional outcomes results in different aspects of motor recovery (Hornby et al., 2005; Heller et al., 2007; Mayr et al., 2007; Hidler et al., 2009; Chang et al., 2012; Krishnan et al., 2012; Nilsson et al., 2014; Kim et al., 2015). With over-ground systems, much of the focus has been on compactness (Farris et al., 2011a), and trajectory (Farris et al., 2011b). It should also be noted that some exoskeletons focus on spinal cord injured patients and have mechanical design and control attributes that can transfer to stroke rehabilitation as well. Seated implementations also have potential for reducing ankle impairment, as well as studying the effects of varied feedback on lower extremity motor learning (Forrester et al., 2011).

Recently, interest has been drawn in the design of interfaces that can use internal body measurements or intentions while walking, such as electroencephalography (EEG) signals (He et al., 2014) or surface electromyography (EMG) signals (Ferris et al., 2006). These types of systems, such as a brain-computer interface (BCI), offer an alternative, internal-based method for accessing information about the human body. The goal of using these implementations is to use this neuro-physiological information to provide control-relevant information for a rehabilitation robot to make decisions considering force and timing for movement. Surface EMG has been used in the control of trajectories of full leg manipulation systems (Kawamoto and Sankai, 2007) and ankle foot systems (Ferris et al., 2005) for rehabilitation. These provided non-FES aided gait, as opposed to earlier methods (Goldfarb et al., 2003) that were mainly designed to just provide significantly better trajectory control and to reduce muscle fatigue when compared to FES-only gait. Current and future implementations of this neuro-physiological information have the challenge of overcoming signal variability, classification algorithm robustness, and quantifiable performance feedback indicators (Tariq et al., 2018). Current advances in EMG and EEG analysis have led to broad applications of this control approach in rehabilitation robotics, however these challenges still require solving for these methods to become viable parts of rehabilitation, especially in exoskeleton and orthosis implementations (Ison and Artemiadis, 2014).

Soft actuation has the advantage of providing a more compliant way to interact with natural human morphology and biomechanics (Ortiz et al., 2017). Some successful robotic exoskeletons have even been redesigned as a version using cable routing instead of rigid links (Hidayah et al., 2018). Utilizing soft robotics techniques, a reduction in size and weight can also be achieved (Jin et al., 2018). As demonstrated in Awad et al. (2017), a low assistance soft exosuit that functions in synchrony with a wearer's paretic limb could facilitate an immediate increase in the paretic ankle's swing phase dorsiflexion and increase in the paretic limb's generation of forward propulsion. These improvements can result in a significant reduction in forward propulsion inter-limb asymmetry and reduced the energy cost of walking in ambulatory individuals after stroke, which is an important factor in both lower- and upper-limb soft rehabilitation robot designs (Xiloyannis et al., 2019). These compliance and comfort based systems have shown considerable advantages over traditional rigid exoskeleton designs, and have shown the ability to have similar beneficial outcomes such as increased foot clearance in stroke patient studies (Di Natali et al., 2019). Soft actuation and interfaces have a very promising future in lower-limb robot-assisted rehabilitation.

A powered leg orthosis applies suitable forces to move the leg on a desired trajectory using an assist as needed force-field controller and linear actuators at hip joint and knee joints in Banala et al. (2007b,a). This approach resists undesirable gait motion and provides assistance toward the desirable motion by applying forces at the foot of the subject (Banala et al., 2009). Furthermore, the work in Banala et al. (2010) showed that subjects with a force-field based control and with visual guidance produced considerable adaptation of their normal gait pattern toward the prescribed gait pattern when compared to a separate group receiving only visual guidance. An assist-as-needed paradigm with visual feedback is also a promising application for force-field based control methods in exoskeletons (Srivastava et al., 2015). Another active ankle-foot orthosis presented in Blaya and Herr (2004) tests the idea of modulating impedance of the orthotic joint throughout the gait cycle to treat drop-foot gait. Implementing an adaptive trajectory control to guide a patient's limb within a desired path (Bortole et al., 2013) allowed a deviation based on torque of interaction between the user and the system. This also used an admittance control strategy that allows the robotic platform to capture the user's movements during assistive training and replicates it during active training. Experimental results show that an exoskeleton can adapt a pre-recorded gait pattern of a specific user that can be adjusted by clinicians, then updated (Bortole et al., 2015) for future experiments.

### 3.4. Treadmill Training

Many of the exoskeleton implementations also make use of a treadmill for training in order to keep certain variables consistent, such as average walking speed. However, treadmill training can be used without use of any direct attachment or robotic device. The treadmill allows for the execution of many walking cycles in a relatively small and controlled space (Hesse, 2008). This allows for any sensors, motion capture camera systems or other data gathering systems to be placed near the subject for local experiments and trials. Training with a split-belt treadmill gives the ability to study of short-term motor adaptations when walking (Skidmore and Artemiadis, 2015), which have been shown to have improved long-term effects in post-stroke gait (Reisman et al., 2012).

### 3.5. Goal-Directed and Task-Oriented Training

Many treadmill-based systems have specialized functions that apply changes to the walking surface. Goal-directed movements that force the subject to produce specific movements can evoke muscle activity that may not be shown during normal, level ground walking. One type of non-conventional surface change used in rehabilitation is speed or direction variation. This is shown by authors in Choi and Bastian (2007) that set both sides of a split-belt treadmill to different speeds or in opposing directions, showing the ability of human motor adaptation. The authors in Choi and Bastian (2007) used a split-belt treadmill to induce motor adaptation by setting both sides of the belt to different speeds and in opposing directions. This technique has been shown to also increase gait speed when coupled with a Virtual Reality (VR) environment (Fung et al., 2006). Furthermore, inducing an unexpected acceleration of the trailing limb can have an increase in propulsive forces, which is a common metric for assessing walking ability (Farrens et al., 2019). This study also allowed the user to actively change the treadmill speed in real time, which has also shown promise of higher walking speed in stroke patients (Ray et al., 2020). Another split-belt treadmill training method that unilaterally changes the walking surface compliance has been shown to provide insight into the role of sensory feedback in perturbed gait, while highlighting mechanisms of inter-leg coordination (Skidmore and Artemiadis, 2016b,a). A change in slope, whether simulated with a tether (Hollerbach et al., 2001), implemented in foot plates (Iwata et al., 2002), or an actual change in level of a treadmill (Eng and Fang Tang, 2011), can be used to manipulate intensity of gait training and give another way to offer task-specific, eccentric therapy (Basso et al., 2018). Stair climbing is another intensive training method that has been tested (Hesse et al., 2010). A goal-directed or task-oriented therapy can be coupled with visual feedback to produce resulting muscle changes through obstacle avoidance or through targeting muscle activation objectives on a screen.

### 3.6. Electrical and Magnetic Stimulation

Instead of implementing robotic systems to interact with the subject, Functional Electrical Stimulation (FES) has been proposed to implement electrical excitation directly onto the muscle. When coordinated, induced muscle contractions can be useful for drop-foot prevention (Peckham and Knutson, 2005). This is used in some exoskeletons for spinal cord injured individuals (Schmitt et al., 2004; Farris et al., 2009a,b; Quintero et al., 2010, 2012; Ha et al., 2012, 2016), and to study the effects of synchronization while walking (Dohring and Daly, 2008). FES may improve the fitness and strength of stroke patients who still have a level of voluntary control (Tong et al., 2006). Moreover, it has been shown to produce positive results when used in conjunction with a treadmill (Hesse et al., 1995).

Transcranial Magnetic Stimulation (TMS) depolarizes cortical nerve membranes and discharges groups of neurons by an induced magnetic field near the cortex of the brain (Lamontagne et al., 2007). The Motor Evoked Potential (MEP) recorded in muscles has been studied in the past (Lotze et al., 2003; Forrester et al., 2006, 2009). This method has been used early on to study H-reflex (Petersen et al., 1998), stretch reflex (Shemmell et al., 2009; Zuur et al., 2009), and transcortical reflexes (Christensen L.O.D. et al., 2000). This disruption of electrical transmissions in the brain is generally considered safe and reversible (O'Dell et al., 2009). TMS and other transcranial stimulations have shown limited use in long-term post-stroke gait rehabilitation, but they can provide new opportunities to study supraspinal mechanisms and cortical activations that might provide useful insight for gait rehabilitation (Lamontagne et al., 2007).

## 4. Targeted Sensorimotor Pathways

Stroke rehabilitation relies on the ability of the brain to recover through neuroplasticity. Neuroplasticity occurs when brain cells regenerate, re-establish, and rearrange neural connections in response to the damage inflicted by a stroke. Specifically on motor rehabilitation, physical therapy that engages sensori-motor mechanisms sparks neuroplasticity, encouraging the brain to correct mental and physical deficits (Morton and Bastian, 2006). This naturally places the third piece of the stroke rehabilitation puzzle: finding, evoking, and manipulating the neural mechanisms that take advantage of the brain's plasticity. Effective rehabilitation techniques maximize this neuroplasticity to achieve an optimal outcome for each patient (Gassert and Dietz, 2018). All rehabilitation methods should use ideas from this category in order to close the gap between neuroscience-based problems and engineering solutions.

### 4.1. Vision

Visual feedback has been utilized as a way provide sensory input to supraspinal mechanisms related to either the subject's position or motion in space. Modalities that have been used in the past focus on displaying spatial feedback (Unluhisarcikli et al., 2011) such as position, trajectory, progress, or statistics about movements, and typically entail moving a mechanical device attached to the subject's limb in order to hit some type of on screen target (Forrester et al., 2013), to maintain desired force (Forrester et al., 2006), or center of rotation (Nalam and Lee, 2019). This encourages patients to improve their movements (Lunenburger et al., 2004) by activation of targeted muscle groups in order to improve functional outcomes. Those methods usually include video game-based therapy methods to enhance visuo-motor coordination while increase patient's engagement (Deutsch et al., 2009). Lately, Virtual Reality (VR) has been proposed as a more engaging and effective way to stimulate visuo-motor pathways and induce plasticity. This is a fast-growing way to implement a Virtual Environment (VE) most commonly by attaching a headset over the eyes, covering the entire visual space of the subject with the virtual environment. With VR, only a program and headset are required to interact with any physical setting, and can be even implemented in tele-rehabilitation training (Deutsch et al., 2007). People with disabilities including stroke show promise of motor learning within virtual environments (Holden, 2005), as well as increasing gait speed (Fung et al., 2006). However, there is a need to better understand the neural mechanisms that validates VR in the stroke rehabilitation field (Fluet and Deutsch, 2013). With a trained therapist, these systems can be used to “monitor, manipulate, and augment the users' interaction with their environment” toward functional recovery (Wade and Winstein, 2011). This has recently been implemented as a method for studying the effects of perturbations during gait over ground (Martelli et al., 2019), and showed promise when coupled with robotic implementations (Boian et al., 2002, 2005; Mirelman et al., 2009). Typically supplementing rehabilitation techniques already used, VR provides an environment that would normally require a real world setting, increasing the complexity in material set up. If a real-world environment is desired, distinguished from virtual reality, augmented reality places animated objects into the real-world environment. This has the advantage of appearing more realistic to the user and removes any disorientation stemming from VR environments. Since this technology is relatively new, implementations for rehabilitation that are coupled with various other physical implementations and interaction goals are still waiting to be discovered.

### 4.2. Audition

Supplementary feedback such as auditory could supplement or replace vision for wearable systems (Roby-Brami and Jarrassé, 2018), but is also used for socially assistive robots. These can use vocal cues to facilitate movement or provide encouragement and discouragement behavior, and when combined with robotic gestures, vocal grammar is an important part of interactions with the real world (Feil-Seifer and Matarić, 2005). Another application of auditory feedback is rhythmic auditory cueing. This is an approach that synchronizes gait to a rhythm to improve gait measures. There is moderate evidence of improved velocity and stride length in stroke patients after gait training with rhythmic auditory cueing (Winstein et al., 2016).

### 4.3. Equilibrioception

The sense of balance is another mechanism that is important for walking that uses visual and auditory feedback, as well as proprioception (Peshkin et al., 2005). The proprioceptive sense includes various muscle afferents with origin in muscle spindles and Golgi Tendon Organs (GTO's). Proprioception is the sense of having a known position of body parts relative to other parts of the body through regulation of the muscle activation amplitude during and in the switch between the gait phases (Rossignol et al., 2006). Because the muscle spindles are in parallel with the muscle, they provide accurate muscle length and velocity feedback through neural channels. Similarly, GTO's are in series with the tendons of the muscles and sense the muscle force. While the patient is performing either a static or dynamic activity the authors in Khan et al. (2018) show a system for posture training to reduce balance abnormalities by providing proprioceptive haptic feedback. Center of pressure, ground reaction forces and center of mass have been proven to be used by the brain during locomotion. Center of pressure is studied in a powered limb orthosis (Goldfarb et al., 2011) for the control interface to offer effective ways of providing sitting, standing, and walking functionality (Matjačić et al., 2018). The interplay of visual and proprioceptive feedback has also been shown through VR systems (Frost et al., 2015) with promising results.

### 4.4. Cutaneous and Haptic Perception

Haptic feedback is growing in popularity as a possible way to stimulate brain plasticity (Poli et al., 2013). The responses elicited during haptic resistance exercises for healthy individuals (Stegall et al., 2017) suggest that this feedback modality could be utilized for rehabilitation. In fact, haptic feedback may even allow for an increase in motor learning when compared to visual based error amplification (Marchal-Crespo et al., 2019). This unique modality has been shown to activate specific brain structures involved in error-processing (Milot et al., 2018). Haptic feedback has been used in lower limb exoskeletons for posture control (Khan et al., 2018) and conveying feedback information about a desired movement (Olenšek et al., 2018). This type of feedback is also useful for training in bedridden patients (Chisholm et al., 2014) and in this case, is especially useful in maintaining patient engagement (Berezny et al., 2019).

### 4.5. Inter-limb Coordination Mechanisms

Human walking requires coordination of muscle activation patterns between both legs, which seems to be achieved by a flexible neuronal coupling at a spinal level, with each limb affecting the behavior of the other (Swinnen et al., 2013). Typically, the initiation of the swing phase of one leg requires the contralateral leg to simultaneously be in the stance phase. This inter-limb coordination has been shown to be supraspinal based on muscle activation latency (Seiterle et al., 2015). From previous works, it is evident that inter-leg coordination in gait is a process that involves multiple feedback channels and processing of those signals in multiple levels (Christensen L. et al., 2000; Kuo, 2002; Dietz, 2003; Grillner, 2003; Nielsen, 2003; Rossignol et al., 2006; Yang and Gorassini, 2006; Choi and Bastian, 2007; Field-Fote and Dietz, 2007; Forrester et al., 2009; Grillner et al., 2008; Guertin, 2009; Norton, 2010; Petersen et al., 2012). Even though hemiparesis is typically seen as unilateral, almost all of the leg function is bilaterally organized through neural circuitry explained by inter-limb coordination (Kautz and Patten, 2005).

Motivated by early studies of upper inter-limb coordination (Dietz and Berger, 1984; Berger et al., 1987; Sparrow et al., 1987; Kelso et al., 1979), quadrupedal inter-limb coordination (Forssberg et al., 1980), running (Whitall, 1989), and intra-limb coordination (Barela et al., 2000; Haddad et al., 2006; Presacco et al., 2012), unilateral treadmill-based perturbations have been used to study contralateral muscle responses (Dietz et al., 1989; Artemiadis and Krebs, 2011a,b; Skidmore and Artemiadis, 2017). Adults show adapted motor patterns of inter-limb coordination when experimented on split-belt treadmills with varying speeds on each side (Reisman et al., 2005). In the context of hemiparetic gait rehabilitation, the study of inter-limb coordination mechanisms might be of great significance. In fact, it has been shown that neural coupling exists in poststroke patients as it does in healthy subjects (Arya and Pandian, 2014) and for the upper limbs as well (Yoon et al., 2010). In studies with poststroke subjects with hemiparesis, it was found that neural decoupling between the lower limbs perturbs the paretic lower limb function (Kautz and Patten, 2005). It has been also shown that forceful interaction with the non-paretic leg elicits involuntary tension of the resting paretic leg where subjects are supine (Poskanzer, 1972). The central controller requires both locomotion patterns from spinal circuits, as well as neural drive through a multitude of descending pathways, such as proprioception (Poppele et al., 2003), that trigger desired gait corrections from various sensory modalities (Frost et al., 2015). Therefore, both the modeling of muscle activations (Skidmore and Artemiadis, 2016c) and mapping of the brain areas that seem to be involved (Debaere et al., 2001) in inter-limb coordination should be delved into further. From these principles, it is evident that understanding the sensorimotor network of inter-limb coordination is of paramount importance toward providing targeted rehabilitation to hemiparesis and improving the quality of life of patients suffering from it.

## 5. Synopsis and Future Directions

The goal of the paper is to consider previous research on robot-assisted rehabilitation through three different perspectives: the goal and type of interaction, the physical implementation, and the sensorimotor pathways targeted by the robotic devices utilized in the past. Combinations of approaches across groups that have not been attempted yet could lead to new approaches with improved outcomes. As new technologies are developed and new neural links to stroke affected patients are found, a increasingly large number of combinations for implementing these discoveries can be made using the proposed categorization. In other words, the categorization method presented allows for future scientists to fill research gaps with a more universal thought process.

**Figure 2:**
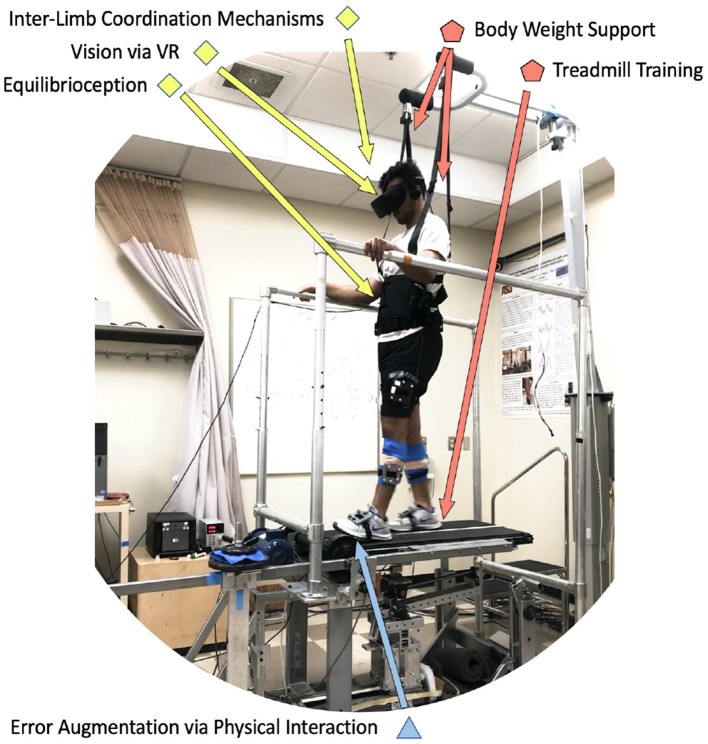
Example of a protocol that uses six components of the proposed organizational chart. A subject wearing a virtual reality headset (*visual*) while walking on a split-belt treadmill with body-weight support (*treadmill training, body weight support*), is experiencing unexpected unilateral walking surface stiffness perturbations (*error augmentation*), which specifically evoke contralateral leg responses (*inter-limb coordination mechanisms*) by disturbing proprioceptive and balance feedback mechanisms (*equilibrioception*).

An example of how this categorization could lead to new methods and approaches is illustrated in Figure 2. In this method, *inter-limb coordination mechanisms* are targeted via *error augmentation* disturbances in experimental setups that include *treadmill training* with *body weight support* through the interplay of *visual* and *equilibrioception*-based feedback. The method above is based on preliminary studies that are already being conducted with a novel device called the variable stiffness treadmill (VST), shown in Figure 2. The VST is a split-belt treadmill with which the compliance of the walking surface can be interactively and dynamically controlled. The VST consists of a spring-loaded lever mounted on a translational linear track that can change the effective stiffness under the foot by moving the linear track. An optical motion capture system monitors the location of the foot in real-time to control the timing of the stiffness perturbations throughout the gait cycle. The effective stiffness of each side/belt of the treadmill can range from 61.7 N/m to theoretically infinite (i.e., rigid walking surface), in 0.13 s. Furthermore, the resolution of the VST stiffness control is about 0.038 N/m (Skidmore et al., 2014, 2015).

According to the protocol followed in this study, a subject wearing a virtual reality headset (*visual*) while walking on a split-belt treadmill with body-weight support (*treadmill training, body weight support*), is experiencing unexpected unilateral walking surface stiffness perturbations (*error augmentation*), that specifically evoke contralateral leg responses (*inter-limb coordination mechanisms*) by disturbing proprioceptive and balance feedback mechanisms (*equilibrioception*). Preliminary results with these tools suggest that muscle and brain activity is evoked on one leg when the stiffness of the walking surface for the other leg is perturbed (Skidmore et al., 2014; Skidmore and Artemiadis, 2015, 2016a,d). The specific outcomes targeted in this study include evoked activity on the ankle muscles of the unperturbed leg, which is very encouraging since this can provide solutions to the problem of drop-foot that most impaired walkers suffer from, and it is the leading cause of after-stroke falls. Recent findings with hemiplegic walkers provide strong evidence that a new method for providing gait rehabilitation could entail evoking activity on the paretic side by introducing unilateral perturbations on the healthy side of hemiplegic walkers (Skidmore and Artemiadis, 2016b, 2017). Therefore, the combination of this type of interactive treadmill system with a variety of specifically timed physical perturbations can significantly broaden our scientific understanding of gait and can open new avenues of research in rehabilitation focusing on the neural and mechanical coupling of the legs, while going beyond the single-leg intervention approaches currently followed.

The above combination of methods and approaches is not unique, and by no means exclusive to what needs to be included in a comprehensive approach in gait rehabilitation. Ideally, selection of approaches ought to be done in the context of a review of gaps and weaknesses found in the empirical evidence. These gaps can be identified using the categorization of approaches this paper introduces. More specifically, Tables 1, 2 provide a comprehensive list of devices and methods used in the past for gait rehabilitation, and how these previous studies can be categorized based on the proposed perspective^1^. Moreover, the tables show if the devices have been tested with patients or not. Although the effectiveness of each approach is not mentioned—and is quite difficult to be assessed and compared across studies—it is important and useful to the future researcher to be able to see how each device or method uses the components of the three categories discussed here, and identify gaps and potential opportunities.

**Table 1:** Literature summary categorized via the proposed organization.

**Device name and Studies**	**Augmentation**	**correction**	**Non-physical**	**Goal/Task-oriented**	**Stimulation**	**Exoskeleton**	**Body weight support**	**Treadmill**	**Footplates**	**Inter-limb coordination**	**Haptic/Cutaneous**	**Equilibrioception**	**Audition**	**Vision**	**Patient tested**
MIT-Skywalker—Artemiadis and Krebs, 2011a,b; Seiterle et al., 2015	•			•			•	•		•	•	•			
Ankle robot—Saglia et al., 2009	•								•		•				
BAR-TM—Matjačić et al., 2018; Olenšek et al., 2018	•							•			•	•		•	•
VST–Barkan et al., 2014; Skidmore et al., 2014; Frost et al., 2015; Skidmore and Artemiadis, 2015; Skidmore et al., 2015; Skidmore and Artemiadis, 2016a,b,c,d, 2017	•			•			•	•		•	•	•		•	•
LOPES—Veneman et al., 2007; Koopman et al., 2013	•	•		•		•	•	•			•	•		•	•
Active/Passive AFO—Barela et al., 2000; Blaya and Herr, 2004; Agrawal et al., 2005; Hwang et al., 2006	•	•		•		•				•	•	•			•
Anklebot—Roy et al., 2007; Forrester et al., 2011, 2013	•	•		•		•					•			•	•
KineAssist—Peshkin et al., 2005	•	•		•			•				•	•			
BWS treadmill—Hesse et al., 1999; Poppele et al., 2003; Haddad et al., 2006; Choi and Bastian, 2007; Field-Fote and Dietz, 2007; Petersen et al., 2012; Presacco et al., 2012; Reisman et al., 2012	•	•		•			•	•		•	•	•			•
NUVABAT—Ding et al., 2010	•	•							•		•	•		•	
Rutgers ankle—Girone et al., 2001; Boian et al., 2002; Deutsch et al., 2007	•	•		•					•		•		•	•	
The gait master—Iwata et al., 2002	•	•		•					•		•	•			
Lokomat—Colombo et al., 2001; Jezernik et al., 2003; Hornby et al., 2005; Lunenburger et al., 2004; Mayr et al., 2007; Heller et al., 2007; Dohring and Daly, 2008; Hidler et al., 2009; Klarner, 2010; Chang et al., 2012; Krishnan et al., 2012	•	•			•	•	•	•			•	•		•	•
ARTHuR—Reinkensmeyer et al., 2003; Emken et al., 2005	•	•				•					•	•			

**Table 2:** Literature summary categorized via the proposed organization (continued).

**Device name and studies**	**Augmentation**	**Correction**	**Non-physical**	**Goal/task-oriented**	**Stimulation**	**Exoskeleton**	**Body weight support**	**Treadmill**	**Footplates**	**Inter-limb coordination**	**Haptic/Cutaneous**	**Equilibrioception**	**Audition**	**Vision**	**Patient tested**
HapticWalker—Schmidt et al., 2005a; Schmidt and Werner, 2007	•	•					•		•		•				•
RMA—Boian et al., 2005	•	•					•		•		•	•		•	
Trunk Support Trainer – Khan et al., 2018	•	•									•	•			
Lambda—Bouri et al., 2009		•							•		•				
Gait Trainer—Hesse et al., 2000; Peurala et al., 2009; Hoölig et al., 2007		•					•		•		•	•			•
Vanderbilt lower limb exoskeleton—Farris et al., 2012; Goldfarb et al., 2003; Ha et al., 2012, 2016; Farris et al., 2014		•		•	•	•					•	•			•
ALEX—Banala et al., 2007a,b, 2009, 2010; Srivastava et al., 2015; Stegall et al., 2017; Hidayah et al., 2018; Jin et al., 2018		•		•		•	•	•			•	•		•	•
HAL—Nilsson et al., 2014; Kawamoto and Sankai, 2007		•		•		•	•				•	•			•
Lokohelp—Freivogel et al., 2008, 2009		•		•			•	•	•		•	•			•
G-EO-Systems Robot—Hesse et al., 2010		•		•			•		•		•				•
ViGGR—Chisholm et al., 2014		•		•							•			•	
JCO—Farris et al., 2009a,b; Quintero et al., 2010		•			•	•					•	•			
Motion Maker—Schmitt et al., 2004		•			•	•					•				
DGO—Colombo et al., 2000		•				•	•	•			•	•			
PAM/POGO—Aoyagi et al., 2007		•				•	•	•			•	•			
WALKBOT—Kim et al., 2015		•				•	•	•			•	•		•	•
WalkTrainer—Bouri et al., 2006; Stauffer, 2009		•				•	•				•	•			•
RGT—Bharadwaj et al., 2005; Bharadwaj and Sugar, 2006		•				•					•				
ANdROS—Unluhisarcikli et al., 2011		•				•					•			•	
Gait Rehabilitation Exoskeleton—Beyl et al., 2008		•				•					•	•			
LLRR– Chen et al., 2009		•					•		•		•	•			
NEUROBike—Monaco et al., 2009		•							•		•				
ROREAS—Gross et al., 2014			•	•										•	•

In conclusion, this paper provides a potential solution to the overwhelming number of gait therapy methods based from the need for utilizing the methods that work and combining them in organized ways to produce new methods, which can potentially have improved outcomes. An example of using this categorization to come up with new methods for rehabilitation, such as perturbation-based approaches using inter-limb coordination mechanisms, is demonstrated. However, this is only one of the possible seeds of new approaches that could sprout from this framework. The authors strongly believe that this new perspective of mixing and matching hardware, procedures, algorithms, and intended neural pathways could lead to more focused research and eventually significant advances in lower-limb robot-assisted stroke rehabilitation.

## Author Contributions

BH and PA contributed conception of the paper. BH organized the database of the references included and wrote the first draft of the manuscript. PA wrote sections of the manuscript. All authors contributed to manuscript revision, read, and approved the submitted version.

### Conflict of Interest

The authors declare that the research was conducted in the absence of any commercial or financial relationships that could be construed as a potential conflict of interest.

## Footnotes

^1^It must be noted that the entries in the Tables 1, 2 are organized in terms of the device used, and how each device makes use of the components discussed in this review paper. When a device has been used in multiple previous works or studies, the cumulative set of components across all papers listed is shown at the tables.
